# Supplementary material for: The triangulation of multimorbidity: A systematic review of primary sleep disorders, hypertension, and psychiatric disorders
Source: PLOS Glob Public Health. 2025 Oct 1;5(10):e0005216. doi: 10.1371/journal.pgph.0005216 (PMC12488021; doi:10.1371/journal.pgph.0005216)
Supplement: S1 File — Risk of Bias evaluation for included studies using the RoBINS-E tool. (PDF) [file pgph.0005216.s002.pdf]

The Risk Of Bias In Non-randomized Studies – of Exposures (ROBINS-E) assessment tool
(for follow-up studies)

Template for completion

Version 24 March 2024

This work is licensed under a Creative Commons Attribution-NonCommercial-NoDerivatives 4.0 International License.

The ROBINS-E tool

At planning stage: list confounding factors and consider appropriateness criteria

P1. List the important confounding factors relevant to all or most studies on this topic. Specify whether these are particular to specific exposures-outcome combinations.

P2. Will the review use the ROBINS-E assessment of appropriateness (important aspects of “study sensitivity”)?

Yes / No

If Yes, complete sections Addressing appropriateness, Parts I and II in Appendix 1.

For each study result: preliminary considerations

A. Specify the result being assessed for risk of bias

A1. Specify the numerical result being assessed

B. Decide whether to proceed with a risk-of-bias assessment

	Response options	Comments
B1. Did the authors make any attempt to control for confounding?	Y / PY / PN / N	
B2. **If N/PN to B1**: Is there sufficient potential for confounding that an unadjusted result should not be considered further?	Y / PY / PN / N	
B3. Was the method of measuring exposure inappropriate?	Y / PY / PN / N	
B4. Was the method of measuring the outcome inappropriate?	Y / PY / PN / N	

If the answer to any of B2, B3 or B4 is 'Yes' or 'Probably yes', the result should be considered to be at very high risk of bias and no further assessment is required. Otherwise, proceed to section C.

C. Specify the analysis in the current study for which results are being assessed for risk of bias

C1. Specify the outcome to which this result relates.

C2. Specify the participant group on which this result was based.

C3 to C8: Describe the exposure measurement(s) used to produce this result.

C3. What is the exposure being measured and how was it measured or assessed?	
C4. Was exposure analysed as a quantitative (rather than a categorical) variable?	Y / PY / PN / N
C5. Did repeated measurements of exposure over time (for each participant) contribute to the analysis that produced this result?	Y / PY / PN / N
C6. **If Y/PY to C5**, was a single estimate of each participant's exposure level derived from the repeated measurements of exposure over time?	NA / Y / PY / PN / N
C7. **If N/PN to C6**, was the analysis based on splitting participants' follow up time according to exposure status and/or magnitude?	NA / Y / PY / PN / N
C8. **If Y/PY to C7**, were changes in exposure status and/or magnitude likely to be related to factors that are predictive of the outcome?	NA / Y / PY / PN / N
C9. **If N/PN to C7**, how were repeat measurements used?	

Y = Yes; PY = Probably yes; PN = Probably no; N = No; NA = Not applicable

C10. Specify the relationship analysed to produce this result. For example, this may be a quadratic relationship of cumulative exposure with the log odds of the outcome, or a risk ratio for the outcome comparing exposed with unexposed individuals.

D: Specify the causal effect of exposure being estimated by this result

D1. Specify the population of interest	Describe eligible participants (to whom the causal effect applies). These may be different from the study participants on whom the result was based (specified in C2). Such differences may give rise to selection biases.	

Specification of the exposure metric of interest

D2. Specify the exposure	This is the factor whose causal effect on the outcome of interest is the subject of the study result being assessed. It may be thought of as the 'true' exposure of interest. It is distinct from the method with which exposure was measured.	
D3. Specify the exposure window	The exposure window of interest is the exposure period for which the result being assessed estimates the effect of exposure on the outcome. Specification of the exposure window is judged by the ROBINS-E user, who should aim to define a window that is both meaningful in answering the review question and broadly in line with when the study measured exposure. Specification should include both the time of onset and period of exposure. For example, it may be lifetime exposure (from birth or from conception), during ages 50-55, the period from first employment in a particular occupation, time from birth to age 10, or during pregnancy.<br><br>The specified exposure window is used to determine whether exposure data adequately reflect exposure during the window. Exposure before the start of the exposure window is addressed during the assessment of risk of bias due to confounding	
D4. Specify how exposure over time	This may, for example, be ever/never exposed, cumulative exposure, average exposure, or peak exposure during the exposure period, for	

should be summarized	each participant. Alternatively, there may be only a single exposure event, or the exposure may be time invariant (such as a genetic variant or family history).	

E. Evaluation of confounding factors

Complete a row for each important confounding factor listed in advance (subsection (i)). In addition, consider any further confounding factors that are either relevant to the setting of this particular study or which the study authors identified as potentially important (subsection (ii)).

"Important" confounding factors are those for which, in the context of this study, adjustment is expected to lead to an important change in the estimated effect of the exposure.

(i) Important confounding factors listed in advance						
Confounding factor	Measured variable(s) for this factor, if any	Was this variable (or were these variables) controlled for in the analysis?<br>(Y / N)	If this confounding factor was controlled for, was it measured validly and reliably by this variable (or these variables)?*<br>(NA / Y / PY / PN / N / NI)	If this confounding factor was not controlled for, is there evidence that controlling for it was unnecessary?**<br>(NA / Y / PY / PN / N)	Is failure to adjust for this confounding factor expected to bias the effect estimate towards benefit or harm of (higher) exposure?***<br>(Benefit of (higher) exposure / Harm of (higher) exposure / Insufficient information available)	Comments

(ii) Additional confounding factors relevant to the setting of this particular study, or identified by study authors and considered to be important, or which were identified since the protocol was written						
Confounding factor	Measured variable(s) for this factor, if any	Was this variable (or were these variables) controlled for in the analysis?<br>(Y / N)	If this confounding factor was controlled for, was it measured validly and reliably by this variable (or these variables)?*<br>(NA / Y / PY / PN / N / NI)	If this confounding factor was not controlled for, is there evidence that controlling for it was unnecessary?**<br>(NA / Y / PY / PN / N)	Is failure to adjust for this confounding factor expected to bias the effect estimate towards benefit or harm of (higher) exposure?*** (Benefit of (higher) exposure / Harm of (higher) exposure / Insufficient information available)	Comments

* “Validity” refers to whether the confounding variable or variables accurately measure the confounding factor, while “reliability” refers to the precision of the measurement (more measurement error means less reliability).

** In the context of a particular study, variables need not be included in the analysis: (a) if they are measured validly and reliably and are not associated with the outcome, conditional on exposure (noting that lack of a statistically significant association is not evidence of a lack of association); (b) if they are measured validly and reliably and are not associated with exposure; (c) if they are measured validly and reliably and adjustment makes no or minimal difference to the estimated effect of the primary parameter; (d) because the confounder was addressed in the study design, for example by restricting to individuals with the same value of the confounder; (e) because a negative control demonstrates that there was unlikely to have been confounding due to this variable or that uncontrolled confounding was likely to be minimal; or (f) because external evidence suggests that controlling for the variable is not necessary in the context of the study being assessed..

For each study: risk of bias assessment

Domain 1: Risk of bias due to confounding

*Domain 1, Variant (a): **If N/PN to C5 or Y/PY to C6 or N/PN to C7** (only baseline confounding needs to be addressed)*

Signalling questions	Response options	Comments
1.1 Did the authors control for all the important confounding factors for which this was necessary?	Y / PY / WN (no, but uncontrolled confounding was probably not substantial) / SN (no, and uncontrolled confounding was probably substantial) / NI	
1.2 **If Y/PY/WN to 1.1**: Were confounding factors that were controlled for (and for which control was necessary) measured validly and reliably by the variables available in this study?	NA / Y / PY / WN (no, but the extent of measurement error in confounding factors was probably not substantial) / SN (no, and the extent of measurement error in confounding factors was probably substantial) / NI	
1.3 **If Y/PY/WN to 1.1**: Did the authors control for any variables after the start of the exposure period being studied that could have been affected by the exposure?	NA / Y / PY / PN / N / NI	
1.4 Did the use of negative controls, or other considerations, suggest serious uncontrolled confounding?	Y / PY / PN / N	
Risk of bias (due to confounding) in the estimated effect of exposure on the outcome	Low risk / Some concerns / High risk / Very high risk	

Signalling questions	Response options	Comments
What is the predicted direction of bias due to confounding?	(Towards benefit of (higher) exposure / Towards harm of (higher) exposure / Insufficient information available)	
Is the risk of bias (due to confounding) sufficiently high, in the context of its likely direction and the magnitude of the estimated exposure effect, to threaten conclusions about whether the exposure has an important effect on the outcome?	Yes / No / Cannot tell	

Y = Yes; PY = Probably yes; PN = Probably no; N = No; SY = Strong yes; WY = Weak yes; SN = Strong no; WN = Weak no; NA = Not applicable; NI = No information

*Domain 1, variant (b): **If Y/PY to C7 and Y/PY to C8** (the analysis was based on splitting participants' follow up time according to exposure status and/or magnitude and changes in exposure status and/or magnitude likely to be related to factors that are predictive of the outcome, so both baseline and time-varying confounding need to be addressed)*

Signalling questions	Response options	Comments
1.1 Did the authors use an analysis method that was appropriate to control for time-varying as well as baseline confounding?	Y / PY / PN / N / NI	
1.2 **If Y/PY to 1.1**: Did the authors control for all the important baseline and time-varying confounding factors for which this was necessary?	NA / Y / PY / WN (no, but uncontrolled confounding was probably not substantial) / SN (no, and uncontrolled confounding was probably substantial) / NI	
1.3 **If Y/PY/WN to 1.2**: Were confounding factors that were controlled for (and for which control was necessary) measured validly and reliably by the variables available in this study?	NA / Y / WN (no, but the extent of measurement error in confounding factors was probably not substantial) / SN (no, and the extent of measurement error in confounding factors was probably substantial) / NI	
1.4 **If N/PN/NI to 1.1**: Did the authors control for time-varying factors or other variables measured after the start of the exposure window being studied?	NA / Y / PY / PN / N / NI	
1.5 Did the use of negative controls, or other considerations, suggest uncontrolled confounding?	Y / PY / PN / N	
Risk of bias (due to confounding) in the estimated effect of exposure on the outcome	Low risk / Some concerns / High risk / Very high risk	

Signalling questions	Response options	Comments
What is the predicted direction of bias due to confounding?	Towards benefit of (higher) exposure / Towards harm of (higher) exposure / Towards null / Away from null / Insufficient information available	
Is the risk of bias (due to confounding) sufficiently high, in the context of its likely direction and the magnitude of the estimated exposure effect, to threaten conclusions about whether the exposure has an important effect on the outcome?	Yes / No / Cannot tell	

Y = Yes; PY = Probably yes; PN = Probably no; N = No; SY = Strong yes; WY = Weak yes; SN = Strong no; WN = Weak no; NA = Not applicable; NI = No information

Domain 2: Risk of bias arising from measurement of the exposure

*Domain 2, Variant (a): **If N/PN to C5** (exposure was measured at a single point in time)*

Signalling questions	Response options	Comments
**Mismeasurement or misclassification of the exposure.**		
**2.1 Does the measured exposure well-characterize the exposure metric specified to be of interest in this study? [*This was specified in the answers to D2, D3 and D4*]**	**Y / PY / WN (no, to a small extent) / SN (no, to a large extent) / NI**	
**2.2 Was the exposure likely to be measured with error, or misclassified?**	**SY (yes, probably a substantial amount) / WY (yes, but probably not a substantial amount) / PN / N / NI**	
**Bias in the estimated effect of exposure arising from mismeasurement or misclassification of the exposure**		
**2.3 If SY/WY to 2.2: Could mismeasurement or misclassification of exposure have been differential (i.e. related to the outcome or risk of the outcome)?**	**NA / SY (yes, to a large extent) / WY (yes, to a small extent) / PN / N / NI**	
**2.4 If SY/WY to 2.2 and N/PN/WY to 2.3: Is non-differential measurement error likely to bias the estimated effect of exposure on outcome?**	**NA / SY (yes, to a large extent) / WY (yes, to a small extent) / PN / N / NI**	
**Risk of bias (arising from measurement of exposure) in the estimated effect of exposure on the outcome**	**Low risk / Some concerns / High risk / Very high risk**	
**What is the predicted direction of bias arising from measurement of exposure?**	**Towards benefit of (higher) exposure / Towards harm of (higher) exposure / Towards null / Away from null / Insufficient information available**	

Signalling questions	Response options	Comments
**Is the risk of bias (arising from measurement of exposure) sufficiently high, in the context of its likely direction and the magnitude of the estimated exposure effect, to threaten conclusions about whether the exposure has an important effect on the outcome?**	**Yes / No / Cannot tell**	

Y = Yes; PY = Probably yes; PN = Probably no; N = No; SY = Strong yes; WY = Weak yes; SN = Strong no; WN = Weak no; NA = Not applicable; NI = No information

*Domain 2, Variant (b): **If Y/PY to C5 and Y/PY to C6** (each individual's exposure level was estimated from measurements made at multiple time points)*

Signalling questions	Response options	Comments
2.1 Does the measured exposure (derived from measurements at multiple time points) well-characterize the exposure metric specified to be of interest in this study? [*This was specified in the answers to D2, D3 and D4*]	Y / PY / WN (no, to a small extent) / SN (no, to a large extent) / NI	
2.2 Was there error in measurement, or misclassification, of the exposure, at each single time point?	SY (yes, probably a substantial amount) / WY (yes, but probably not a substantial amount) / PN / N / NI	
2.3 If SY/WY to 2.2: Could mismeasurement or misclassification of exposure have been differential (i.e. related to the outcome or risk of the outcome)?	NA / SY (yes, to a large extent) / WY (yes, to a small extent) / PN / N / NI	
2.4 If SY/WY to 2.2 and N/PN/WY to 2.3: Is the nature of the (non-differential) measurement error likely to bias the estimated effect of exposure on outcome?	NA / SY (yes, to a large extent) / WY (yes, to a small extent) / PN / N / NI	
Risk of bias (arising from measurement of exposure) in the estimated effect of exposure on the outcome	Low risk / Some concerns / High risk / Very high risk	
What is the predicted direction of bias arising from measurement of exposure?	Towards benefit of (higher) exposure / Towards harm of (higher) exposure / Towards null / Away from null / Insufficient information available	
Is the risk of bias (arising from measurement of exposure) sufficiently high, in the context of its likely direction and the magnitude of the estimated exposure effect, to threaten conclusions about	Yes / No / Cannot tell	

Signalling questions	Response options	Comments
**whether the exposure has an important effect on the outcome?**		

Y = Yes; PY = Probably yes; SN = Strong no; WN = Weak no; NA = Not applicable; NI = No information

*Domain 2, Variant (c): **If Y/PY to C5, N/PN to C6 and Y/PY to C7** (the analysis was based on splitting participants' follow up time according to exposure status and/or magnitude):*

Signalling questions	Response options	Comments
**2.1 Does the measured exposure (including changes over time) well-characterize the exposure metric specified to be of interest in this study? [*This was specified in the answers to D2, D3 and D4*]**	**Y / PY / WN (no, to a small extent) / SN (no, to a large extent) / NI**	
**2.2 Was there error in measurement, or misclassification, of the exposure, at each single time point?**	**SY (yes, probably a substantial amount) / WY (yes, but probably not a substantial amount) / PN / N / NI**	
**2.3 If SY/WY to 2.2: Could mismeasurement or misclassification of exposure have been differential (i.e. related to the outcome or risk of the outcome)?**	**NA / SY (yes, to a large extent) / WY (yes, to a small extent) / PN / N / NI**	
**2.4 If SY/WY to 2.2 and N/PN/WY to 2.3: Is the nature of the (non-differential) measurement error likely to bias the estimated effect of exposure on outcome?**	**NA / SY (yes, to a large extent) / WY (yes, to a small extent) / PN / N / NI**	
**Risk of bias (arising from measurement of exposure) in the estimated effect of exposure on the outcome**	**Low risk / Some concerns / High risk / Very high risk**	
**What is the predicted direction of bias arising from measurement of exposure?**	**Towards benefit of (higher) exposure / Towards harm of (higher) exposure / Towards null / Away from null / Insufficient information available**	
**Is the risk of bias (arising from measurement of exposure) sufficiently high, in the context of its likely direction and the magnitude of the estimated exposure effect, to threaten conclusions about**	**Yes / No / Cannot tell**	

**whether the exposure has an important effect on the outcome?**		

Y = Yes; PY = Probably yes; SN = Strong no; WN = Weak no; NA = Not applicable; NI = No information

Domain 3: Risk of bias in selection of participants into the study (or into the analysis)

Signalling questions	Response options	Comments
3.1 Did follow-up begin at (or close to) the start of the exposure window for most participants? [*The exposure window is specified in D3*]	Y / PY / PN / N / NI	
3.2 **If N/PN to 3.1**: Is the effect of exposure likely to be constant over the period of follow up analysed?	NA / Y / PY / PN / N / NI	
3.3 Was selection of participants into the study (or into the analysis) based on participant characteristics observed after the start of the exposure window being studied? [*The exposure window is specified in D3*]	Y / PY / PN / N / NI	
3.4 **If Y/PY to 3.3**: Were these characteristics likely to be influenced by exposure or a cause of exposure?	NA / Y / PY / PN / N / NI	
3.5 **If Y/PY to 3.4**: Were these characteristics likely to be influenced by the outcome or a cause of the outcome?	NA / Y / PY / PN / N / NI	
3.6 **If N/PN to 3.2 or Y/PY to 3.5**: Is it likely that the analysis corrected for all of the potential selection biases identified in A and B above?	NA / Y / PY / PN / N / NI	
3.7 **If N/PN to 3.2 or Y/PY to 3.5**: Did sensitivity analyses demonstrate that the likely impact of the potential selection biases identified in A or B above was minimal?	NA / Y / PY / WN (no, there were no sensitivity analyses or there is evidence of some impact) / SN (no, there is evidence of substantial impact)	
Risk of bias (due to selection of participants into the study) in the estimated effect of exposure on the outcome	Low risk / Some concerns / High risk / Very high risk	
What is the predicted direction of bias<br><br>due to selection of participants into the study?	Towards benefit of (higher) exposure / Towards harm of (higher) exposure / Towards null / Away from null /	

Signalling questions	Response options	Comments
	Insufficient information available	
Is the risk of bias (due to selection of participants into the study) sufficiently high, in the context of its likely direction and the magnitude of the estimated exposure effect, to threaten conclusions about whether the exposure has an important effect on the outcome?	Yes / No / Cannot tell	

Y = Yes; PY = Probably yes; PN = Probably no; N = No; SN = Strong no; WN = Weak no; NA = Not applicable; NI = No information

Domain 4: Risk of bias due to post-exposure interventions

Signalling questions	Response options	Comments
4.1 Were there post-exposure interventions that were influenced by prior exposure during the follow-up period?	Y / PY / PN / N / NI	
4.2 **If Y/PY to 4.1**: Is it likely that the analysis corrected for the effect of post-exposure interventions that were influenced by prior exposure?	NA / Y / PY / PN / N / NI	
Risk of bias (due post-exposure interventions) in the estimated effect of exposure on the outcome	Low risk / Some concerns / High risk / Very high risk	
What is the predicted direction of bias due to confounding?	Towards benefit of (higher) exposure / Towards harm of (higher) exposure / Towards null / Away from null / Insufficient information available	
Is the risk of bias (due post-exposure interventions) sufficiently high, in the context of its likely direction and the magnitude of the estimated exposure effect, to threaten conclusions about whether the exposure has an important effect on the outcome?	Yes / No / Cannot tell	

Y = Yes; PY = Probably yes; PN = Probably no; N = No; NA = Not applicable; NI = No information

Domain 5: Risk of bias due to missing data

Signalling questions	Response options	Comments
5.1 Were complete data on exposure status available for all, or nearly all, participants?	Y / PY / PN / N / NI	
5.2 Were complete data on the outcome available for all, or nearly all, participants?	Y / PY / PN / N / NI	
5.3 Were complete data on confounding variables available for all, or nearly all, participants?	Y / PY / PN / N / NI	
5.4 **If N/PN/NI to 5.1, 5.2 or 5.3**: Is the result based on a complete case analysis?	NA / Y / PY / PN / N / NI	
5.5 **If Y/PY/NI**: Was exclusion from the analysis because of missing data (in exposure, confounders or the outcome) likely to be related to the true value of the outcome?	NA / SY (Yes, strongly related) / WY (Yes, but not strongly related) / PN / N / NI	
5.6 **If N/PN to 5.5**: Were all or most predictors of missingness (in exposure, confounders or the outcome) included in the analysis model?	NA / SY (Yes, for sure) / WY (Yes, mostly or probably) / PN / N / NI	
5.7 **If N/PN to 5.4**: Was the analysis based on imputing missing values?	NA / Y / PY / PN / N	
5.8 **If Y/PY to 5.7**: Was imputation performed appropriately?	NA / Y / PY / WN (no, but not leading to substantial bias) / SN (no, such that bias would not be substantially reduced) / NI	
5.9 **If N/PN to 5.7**: Was an appropriate alternative method used to correct for bias due to missing data?	NA / Y / PY / WN (no, but not leading to substantial bias) / SN (no, such that bias would	

Signalling questions	Response options	Comments
	not be substantially reduced) / NI	
5.10 **If PN/N/NI to 5.1, 5.2 or 5.3**: Is there evidence that the result was not biased by missing data?	NA / Y / PY / PN / N	
Risk of bias (due to missing data) in the estimated effect of exposure on the outcome	Low risk / Some concerns / High risk / Very high risk	
What is the predicted direction of bias due to missing data?	Towards benefit of (higher) exposure / Towards harm of (higher) exposure / Towards null / Away from null / Insufficient information available	
Is the risk of bias (due to missing data) sufficiently high, in the context of its likely direction and the magnitude of the estimated exposure effect, to threaten conclusions about whether the exposure has an important effect on the outcome?	Yes / No / Cannot tell	

Y = Yes; PY = Probably yes; PN = Probably no; N = No; SY = Strong yes; WY = Weak yes; NA = Not applicable; NI = No information

Domain 6: Risk of bias arising from measurement of the outcome

Signalling questions	Response options	Comments
6.1 Could measurement or ascertainment of the outcome have differed between exposure groups or levels of exposure?	Y / PY / PN / N / NI	
6.2 Were outcome assessors aware of study participants' exposure history?	Y / PY / PN / N / NI	
6.3 **If Y/PY/NI to 6.2**: Could assessment of the outcome have been influenced by knowledge of participants' exposure history?	NA / SY (yes, to a large extent) / WY (yes, to a small extent) / PN / N / NI	
Risk of bias (arising from measurement of outcomes) in the estimated effect of exposure on the outcome	Low risk / Some concerns / High risk / Very high risk	
What is the predicted direction of bias arising from measurement of outcomes?	Towards benefit of (higher) exposure / Towards harm of (higher) exposure / Towards null / Away from null / Insufficient information available	
Is the risk of bias (arising from measurement of outcomes) sufficiently high, in the context of its likely direction and the magnitude of the estimated exposure effect, to threaten conclusions about whether the exposure has an important effect on the outcome?	Yes / No / Cannot tell	

Y = Yes; PY = Probably yes; PN = Probably no; N = No; SY = Strong yes; WY = Weak yes; NA = Not applicable; NI = No information

Domain 7: Risk of bias in selection of the reported result

Signalling questions	Response options	Comments
7.1 Was the result reported in accordance with an available, pre-determined analysis plan?	Y / PY / PN / N / NI	
7.2 **If N/PN/NI to 7.1**: Is the reported effect estimate likely to be selected, based on desirability of the magnitude (or statistical significance) of the estimated effect of exposure on outcome, from multiple *exposure measurements* within the exposure domain?	NA / Y / PY / PN / N / NI	
7.3 Is the reported effect estimate likely to be selected, based on desirability of the magnitude (or statistical significance) of the estimated effect of exposure on outcome, from multiple *outcome measurements* within the outcome domain?	Y / PY / PN / N / NI	
7.4 Is the reported effect estimate likely to be selected, based on desirability of the magnitude (or statistical significance) of the estimated effect of exposure on outcome, from multiple *analyses* of the exposure-outcome relationship?	Y / PY / PN / N / NI	
7.5 Is the reported effect estimate likely to be selected, based on the basis of desirability of the results (e.g. statistical significance), from different *subgroups*?	Y / PY / PN / N / NI	
Risk of bias (due to selection of the reported result) in the estimated effect of exposure on the outcome	Low risk / Some concerns / High risk / Very high risk	
What is the predicted direction of bias due to selection of the reported result?	Towards benefit of (higher) exposure / Towards harm of (higher) exposure / Towards null / Away from null /	

Signalling questions	Response options	Comments
	Insufficient information available	
Is the risk of bias (due to selection of the reported result) sufficiently high, in the context of its likely direction and the magnitude of the estimated exposure effect, to threaten conclusions about whether the exposure has an important effect on the outcome?	Yes / No / Cannot tell	

Y = Yes; PY = Probably yes; PN = Probably no; N = No; NA = Not applicable; NI = No information

Overall risk of bias

	Response options	Comments
Overall risk of bias	Low risk of bias except for concerns about uncontrolled confounding / Some concerns / High risk / Very high risk	
What is the predicted direction of bias?	Towards benefit of (higher) exposure / Towards harm of (higher) exposure / Towards null / Away from null / Insufficient information available	
Is the overall risk of bias sufficiently high, in the context of its likely direction and the magnitude of the estimated exposure effect, to threaten conclusions about whether the exposure has an important effect on the outcome?	Yes / No / Cannot tell	

This work is licensed under a Creative Commons Attribution-NonCommercial-NoDerivatives 4.0 International License.
