# Supplementary material for: The triangulation of multimorbidity: A systematic review of primary sleep disorders, hypertension, and psychiatric disorders
Source: PLOS Glob Public Health. 2025 Oct 1;5(10):e0005216. doi: 10.1371/journal.pgph.0005216 (PMC12488021; doi:10.1371/journal.pgph.0005216)
Supplement: Attachment — Submitted filename: Comment.pdf [file pgph.0005216.s005.pdf]

Comment

1. Thank you for responding to our previous requests. We note that you have responded with the following statement in regards to the completed quality assessment:

   "We have now included in the supplementary materials presenting the completed risk of bias and quality/certainty assessments for each study and outcome, following the Cochrane risk-of-bias tool."

   However, we note that this is not included in your supplementary materials. Please check that all relevant files and figures have been uploaded and re-upload any if necessary.

   Thank you for your careful review and for bringing this to our attention. We have included the results of the risk-of-bias assessment in the Excel sheet for the included studies. Additionally, the risk-of-bias tool has been uploaded in checklist format as part of the supplementary material.
