# Supplementary material for: The triangulation of multimorbidity: A systematic review of primary sleep disorders, hypertension, and psychiatric disorders
Source: PLOS Glob Public Health. 2025 Oct 1;5(10):e0005216. doi: 10.1371/journal.pgph.0005216 (PMC12488021; doi:10.1371/journal.pgph.0005216)
Supplement: Attachment — Submitted filename: Editor and reviewer Comments.docx [file pgph.0005216.s006.docx]

**Additional Editor Comments (if provided):**

Figure 1 is pixelated

**Thank you for pointing this out. We have replaced Figure 1 with a high-resolution version in the revised manuscript to ensure better clarity.**

I appreciate the authors for conducting the meticulous systematic review. The article needs further revision to proceed to next stage.

**We thank the Editor for their feedback and appreciation of our work. We acknowledge that further revision is needed and have carefully revised the manuscript accordingly.**

Authors need to elaborate in introduction and discussion on the relevance of this article and the scope of PGPH Journal. The public health implications of insomnia, hypertension and Common mental illnesses needs to be mentioned.

**We thank the reviewer for this constructive comment. In response, we have revised both the Introduction and Discussion sections to more clearly highlight the relevance of our study within the scope of Global Public Health journal. In the Introduction, we elaborated on the significant public health burden associated with insomnia, hypertension, and common mental illnesses such as depression and anxiety. In the Discussion, we further addressed the public health implications of these conditions, emphasizing their impact on population well-being and healthcare systems.**

Funnel plot can be added to describe any publication bias
Odds ratios cab be presented in forest plot format

**Thank you for this valuable suggestion. We attempted to generate a funnel plot and a forest plot for the odds ratios; however, due to the considerable heterogeneity among the included studies—in terms of design, populations, and reported outcomes—we were unable to establish a consistent and comparable model. As a result, it was not feasible to produce a meaningful funnel plot or a summary forest plot. We have mentioned this limitation in the revised manuscript.**

**Reviewers' comments:**

**We thank the reviewer for their time and for reviewing our manuscript.**

[NOTE: If reviewer comments were submitted as an attachment file, they will be attached to this email and accessible via the submission site. Please log into your account, locate the manuscript record, and check for the action link "View Attachments". If this link does not appear, there are no attachment files.]
