# Supplementary material for: The triangulation of multimorbidity: A systematic review of primary sleep disorders, hypertension, and psychiatric disorders
Source: PLOS Glob Public Health. 2025 Oct 1;5(10):e0005216. doi: 10.1371/journal.pgph.0005216 (PMC12488021; doi:10.1371/journal.pgph.0005216)
Supplement: Attachment — Submitted filename: Reviewer comments.docx [file pgph.0005216.s007.docx]

**We would like to thank the editor and reviewers for their careful review of our manuscript entitled. We appreciate the insightful comments and constructive suggestions, which have helped us to improve the quality and clarity of our work.**

**Please find below our point-by-point responses to each of the comments**

Journal Requirements:

If the reviewer comments include a recommendation to cite specific previously published works, please review and evaluate these publications to determine whether they are relevant and should be cited. There is no requirement to cite these works unless the editor has indicated otherwise.

**Thank you for your suggestion. We have carefully reviewed the previous reviewer comments. However, we did not find any specific references suggested for citation. As a result, no new references were added in response to this point.**

Please review your reference list to ensure that it is complete and correct. If you have cited papers that have been retracted, please include the rationale for doing so in the manuscript text, or remove these references and replace them with relevant current references. Any changes to the reference list should be mentioned in the rebuttal letter that accompanies your revised manuscript. If you need to cite a retracted article, indicate the article’s retracted status in the References list and also include a citation and full reference for the retraction notice.

**Thank you for your comment. We have thoroughly reviewed the reference list to ensure its completeness and accuracy. No retracted articles were identified among the cited references. Accordingly, no changes were necessary in this regard.**

Additional Editor Comments (if provided):

Thank you for addressing all queries promptly. Kindly note that you have mentioned all studies in the table and yet the citation list is too long. Please be succinct.

**Thank you for your comment. We have reviewed and revised the reference list to ensure conciseness wherever possible. However, in accordance with PRISMA and Cochrane guidelines, all studies included in the systematic review table must be cited in the reference list. Moreover, these studies are also referenced in the main text. Therefore, while we have made efforts to shorten the reference list, we were unable to remove these specific citations without compromising reporting standards.**

[Note: HTML markup is below. Please do not edit.]

Reviewers' comments:

**We thank the reviewer for their time and consideration. As no specific comments were provided, no changes were made in response to this section.**
